# 1,8-Bis(4-fluoro­benzo­yl)naphthalen-2,7-diyl dimethane­sulfonate

**DOI:** 10.1107/S1600536813006788

**Published:** 2013-03-16

**Authors:** Daichi Hijikata, Rei Sakamoto, Katsuhiro Isozaki, Noriyuki Yonezawa, Akiko Okamoto

**Affiliations:** a Department of Organic and Polymer Materials Chemistry, Tokyo University of Agriculture & Technology (TUAT), Koganei, Tokyo 184-8588, Japan; b International Research Center for Elements Science, Institute for Chemical Research, Kyoto University, Gokasho, Uji, Kyoto 611-0011, Japan

## Abstract

The mol­ecule of the title compound, C_26_H_18_F_2_O_8_S_2_, lies across a crystallographic twofold rotation axis. The benzene rings of the 4-fluorobenzoyl groups make dihedral angles of 78.93 (12)° with the naphthalene ring system. An intra­molecular C—H⋯π inter­action occurs. In the crystal, a number of C—H⋯O inter­actions link the mol­ecules, forming a three-dimensional structure.

## Related literature

For electrophilic aromatic aroylation of the naphthalene core, see: Okamoto & Yonezawa (2009 ▶); Okamoto *et al.* (2011 ▶). For the crystal structures of closely related compounds, see: Watanabe *et al.* (2010 ▶); Tsumuki *et al.* (2011 ▶); Hijikata *et al.* (2010 ▶, 2012 ▶); Sasagawa *et al.* (2013 ▶).
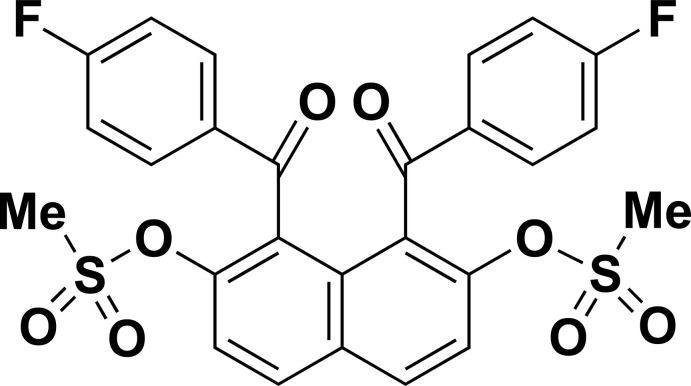


## Experimental

### 

#### Crystal data

C_26_H_18_F_2_O_8_S_2_
*M* *_r_* = 560.52
Monoclinic, 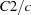

*a* = 7.376 (3) Å
*b* = 16.468 (7) Å
*c* = 20.075 (9) Å
β = 96.123 (6)°
*V* = 2424.4 (19) Å^3^
*Z* = 4
Mo *K*α radiation
μ = 0.29 mm^−1^
*T* = 173 K
0.10 × 0.10 × 0.05 mm

#### Data collection

Rigaku Saturn70 diffractometer
Absorption correction: numerical (*NUMABS*; Higashi, 1999 ▶) *T*_min_ = 0.945, *T*_max_ = 0.945
8755 measured reflections
2360 independent reflections
1617 reflections with *I* > 2σ(*I*)
*R*_int_ = 0.074

#### Refinement

*R*[*F* ^2^ > 2σ(*F* ^2^)] = 0.053
*wR*(*F* ^2^) = 0.120
*S* = 1.03
2360 reflections
173 parameters
H-atom parameters constrained
Δρ_max_ = 0.29 e Å^−3^
Δρ_min_ = −0.33 e Å^−3^

### 

Data collection: *CrystalClear* (Rigaku/MSC, 2006 ▶); cell refinement: *CrystalClear*; data reduction: *CrystalClear*; program(s) used to solve structure: *Il Milione* (Burla *et al.*, 2007 ▶); program(s) used to refine structure: *SHELXL97* (Sheldrick, 2008 ▶); molecular graphics: *ORTEPIII* (Burnett & Johnson, 1996 ▶); software used to prepare material for publication: *SHELXL97*.

## Supplementary Material

Crystal structure: contains datablock(s) I, global. DOI: 10.1107/S1600536813006788/su2570sup1.cif

Structure factors: contains datablock(s) I. DOI: 10.1107/S1600536813006788/su2570Isup2.hkl

Supplementary material file. DOI: 10.1107/S1600536813006788/su2570Isup3.cml

Additional supplementary materials:  crystallographic information; 3D view; checkCIF report

## Figures and Tables

**Table 1:** Hydrogen-bond geometry (Å, °) *Cg* is the centroid of the C8–C13 ring.

*D*—H⋯*A*	*D*—H	H⋯*A*	*D*⋯*A*	*D*—H⋯*A*
C14—H14*A*⋯*Cg*	0.98	2.87	3.805 (4)	160
C14—H14*B*⋯O1^i^	0.98	2.45	3.399 (4)	163
C14—H14*C*⋯O4^ii^	0.98	2.46	3.304 (4)	144
C12—H12⋯O4^iii^	0.95	2.50	3.285 (4)	140
C4—H4⋯O1^iv^	0.95	2.54	3.415 (4)	153

Symmetry codes: (i) 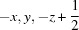; (ii) 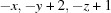; (iii) 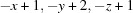; (iv) 
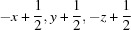
.

**Fig. 1.**
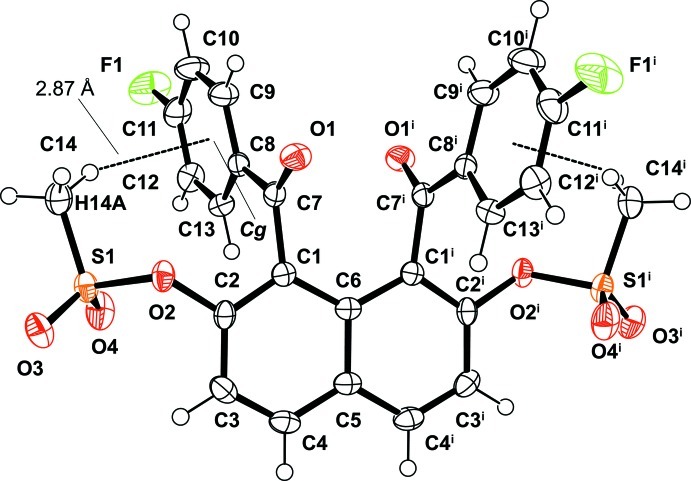
The molecular structure of title molecule, with atom labelling. showing The displacement ellipsoids are drawn at the 50% probability level. The intramolecular C—H···π interactions are shown as a dashed lines (see Table 1 for details; symmetry code: (i) -x+1, y, -z+1/2).

**Fig. 2.**
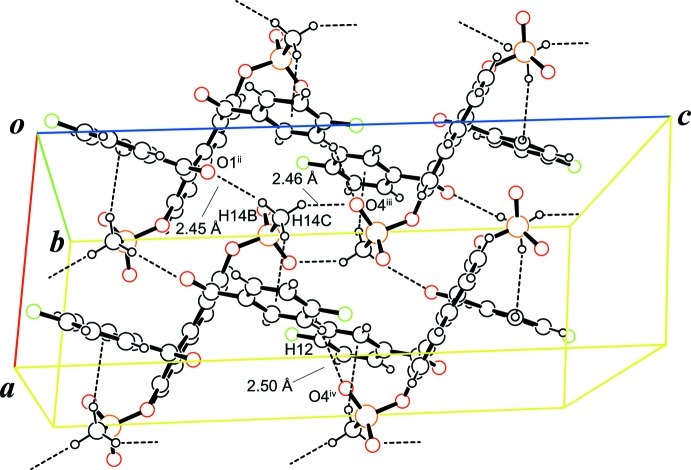
A partial crystal packing diagram of title compound. The C—H···O interactions are shown as dashed lines (see Table 1 for details).

## supplementary crystallographic information

### Comment

In the course of our study on electrophilic aromatic aroylation of 2,7-dimethoxynaphthalene, *peri*-aroylnaphthalene compounds have proven to be formed regioselectively with the aid of suitable acidic mediators (Okamoto & Yonezawa, 2009; Okamoto *et al.*, 2011). Under these circumstances, the authors have stimulated the X-ray crystal structural study of 1,8-diaroylated 2,7-dimethoxynaphthalene analogues exemplified by 1,8-bis(4-fluorobenzoyl)-2,7-dimethoxynaphthalene [(2,7-dimethoxynaphthalene-1,8-diyl)bis(4-fluorophenyl)dimethanone; Watanabe *et al.*, 2010] and 2,7-dimethoxy-1,8-bis(2-naphthoyl)naphthalene {[2,7-dimethoxy-8-(2-naphthoyl)naphthalen-1-yl](naphthalen-2-yl)methanone; Tsumuki *et al.*, 2011}.

Accordingly, to the best of our knowledge, these molecules have essentially the same non-coplanar features, namely: The aroyl groups at the 1,8-positions of the naphthalene ring are bonded in an almost perpendicular fashion and oriented in opposite directions (*anti*-orientation), but the benzene ring moieties of the aroyl groups tilt slightly toward the *exo* sides of the naphthalene ring. Recently, the authors have described the crystal structures of 1,8-diaroylated 2,7-dimethoxynaphthalene analogues, in which the two aroyl groups are situated in the same direction (*syn*-orientation), that is, 2,7-dimethoxy-1,8-bis(4-phenoxybenzoyl)naphthalene (Hijikata *et al.*, 2010) and 2,7-dimethoxy-1,8-bis(4-isopropoxybenzoyl)naphthalene [{2,7-dimethoxy-8-[4-(propan-2-yloxy)benzoyl]naphthalen-1-yl}[4-(propan-2-yloxy)phenyl]methanone; Sasagawa *et al.*, 2013].

The authors then investigated the correlation between the aroyl groups and the neighbouring groups in the spatial organization of 1,8-diaroylnaphthalenes. In the crystal structure of 1,8-bis(4-fluorobenzoyl)-2,7-diphenoxynaphthalene [(4-fluorophenyl)[8-(4-fluorobenzoyl)-2,7-diphenoxynaphthalen-1-yl]methanone; Hijikata *et al.*, 2012], two phenoxy groups are asymmetrically situated with respect to the adjacent aroyl groups. One phenoxy group is horizontal to the aroyl group, whereas another phenoxy group leans toward the naphthalene ring. As a part of our continuous study on the molecular structures of this kind of homologous molecules, the crystal structure of title compound is presented herein.

The molecule of the title compound lies across a crystallographic 2-fold axis, Fig. 1, so that the asymmetric unit contains one-half of the molecule. Thus, the two aroyl groups are situated in an *anti* orientation and twisted away from the naphthalene ring. The benzene ring of the aroyl group make a dihedral angle of 78.93 (12) ° with the naphthalene ring. The dihedral angle between the benzene rings of the aroyl groups is 21.55 (15) °. There are two intramolecular C—H···π interactions in the molecule involving one methyl H atom (H14A) of the methanesulfonyl group and the phenyl ring of the 4-fluorobenzoyl group (Fig. 1 and Table 1).

In the crystal, the ketonic carbonyl oxygen atom (O1) and the sulfonyl oxygen atom (O4) are involved in C—H···O interactions (Fig. 2 and Table 1). These interactions contribute to the stabilization of the packing and lead to the formation a three-dimensional structure (Fig. 2 and Table 1).

### Experimental

1,8-bis(4-fluorobenzoyl)-2,7-dihydroxynaphthalene (1.0 mmol, 404 mg), methanesulfonyl chloride (2.4 mmol, 487 mg), pyridine (10.0 mmol, 791 mg), and methylene chloride (2.5 ml) were placed in a 10 ml flask. The mixture was stirred at room temperature for 24 h. After the reaction, the mixture was extracted with CHCl_3_. The combined extracts were washed with 2 *M* aqueous HCl followed by washing with brine. The organic layers thus obtained were dried over anhydrous MgSO_4_. The solvent was removed under reduced pressure to give cake. The crude product was purified by recrystallization from AcOEt–hexane (*v*/*v*= 2:1) [55% isolated yield; M.p. 507.4 K], giving colourless block-like crystals. Spectroscopic data for the title compound are available in the archived CIF.

### Refinement

All H atoms were placed in calculated positions and treated as riding on their parent atoms: C—H = 0.95 (aromatic C—H), 0.98 (methyl) and *U*_iso_(H) = 1.2*U*_eq_ (aromatic C, methyl C). The positions of methyl H atoms were rotationally optimized.

### Crystal data

**Table:**

C_26_H_18_F_2_O_8_S_2_	*F*(000) = 1152
*M**_r_* = 560.52	*D*_x_ = 1.536 Mg m^−^^3^
Monoclinic, *C*2/*c*	Mo *K*α radiation, λ = 0.71070 Å
Hall symbol: -C 2yc	Cell parameters from 2840 reflections
*a* = 7.376 (3) Å	θ = 2.7–31.5°
*b* = 16.468 (7) Å	µ = 0.29 mm^−^^1^
*c* = 20.075 (9) Å	*T* = 173 K
β = 96.123 (6)°	Block, colorless
*V* = 2424.4 (19) Å^3^	0.10 × 0.10 × 0.05 mm
*Z* = 4	

### Data collection

**Table:**

Rigaku Saturn70 diffractometer	2360 independent reflections
Radiation source: rotating anode	1617 reflections with *I* > 2σ(*I*)
Graphite monochromator	*R*_int_ = 0.074
Detector resolution: 7.314 pixels mm^-1^	θ_max_ = 26.0°, θ_min_ = 3.0°
ω scans	*h* = −9→9
Absorption correction: numerical (*NUMABS*; Higashi, 1999)	*k* = −20→16
*T*_min_ = 0.945, *T*_max_ = 0.945	*l* = −17→24
8755 measured reflections	

### Refinement

**Table:**

Refinement on *F*^2^	Primary atom site location: structure-invariant direct methods
Least-squares matrix: full	Secondary atom site location: difference Fourier map
*R*[*F*^2^ > 2σ(*F*^2^)] = 0.053	Hydrogen site location: inferred from neighbouring sites
*wR*(*F*^2^) = 0.120	H-atom parameters constrained
*S* = 1.03	*w* = 1/[σ^2^(*F*_o_^2^) + (0.0523*P*)^2^ + 1.1088*P*] where *P* = (*F*_o_^2^ + 2*F*_c_^2^)/3
2360 reflections	(Δ/σ)_max_ < 0.001
173 parameters	Δρ_max_ = 0.29 e Å^−^^3^
0 restraints	Δρ_min_ = −0.33 e Å^−^^3^

### Special details

**Table:**

Experimental. Spectroscopic data for the title compound:^1^H NMR δ (500 MHz, DMSO-*d*_6_, 373 K): 3.05(6*H*, s), 7.16(4*H*, dd, *J*_H—H_ = 8.6 Hz, *J*_H—F_ = 8.6 Hz, Ar), 7.58(4*H*, dd, *J*_H—H_ = 8.6 Hz, *J*_H—F_ = 5.7 Hz, Ar), 7.78(2*H*, d, *J* = 9.2 Hz) 8.41(2*H*, d, *J* = 9.2 Hz) p.p.m.^13^C NMR δ (125 MHz, DMSO-*d*_6_, 373 K): 38.27, 114.98(d, ^2^*J*_C—F_ = 22.7 Hz), 120.49 127.19, 127.87, 130.29, 131.66(d, ^3^*J*_C—F_ = 9.5 Hz), 132.46, 133.68(d, ^4^*J*_C—F_ = 2.3 Hz), 145.61, 164.88(d, ^1^*J*_C—F_ = 253.1 Hz), 191.08 p.p.m.IR (KBr): 1673 (C=O), 1594, 1505 (Ar, naphthalene), 1354, 1169 (–SO_2_–) cm^-1^.HRMS (*m/z*): [*M* + H]^+^ calcd for C_26_H_19_F_2_O_8_S_2_, 561.0489 found, 561.0459
Geometry. All e.s.d.'s (except the e.s.d. in the dihedral angle between two l.s. planes) are estimated using the full covariance matrix. The cell e.s.d.'s are taken into account individually in the estimation of e.s.d.'s in distances, angles and torsion angles; correlations between e.s.d.'s in cell parameters are only used when they are defined by crystal symmetry. An approximate (isotropic) treatment of cell e.s.d.'s is used for estimating e.s.d.'s involving l.s. planes.
Refinement. Refinement of *F*^2^ against ALL reflections. The weighted *R*-factor *wR* and goodness of fit *S* are based on *F*^2^, conventional *R*-factors *R* are based on *F*, with *F* set to zero for negative *F*^2^. The threshold expression of *F*^2^ > σ(*F*^2^) is used only for calculating *R*-factors(gt) *etc*. and is not relevant to the choice of reflections for refinement. *R*-factors based on *F*^2^ are statistically about twice as large as those based on *F*, and *R*- factors based on ALL data will be even larger.

### Fractional atomic coordinates and isotropic or equivalent isotropic displacement parameters (Å^2^)

**Table:**

	*x*	*y*	*z*	*U*_iso_*/*U*_eq_	
S1	−0.00621 (10)	1.00592 (5)	0.39209 (3)	0.0260 (2)	
F1	0.5336 (3)	0.76896 (12)	0.54084 (9)	0.0563 (6)	
O1	0.2861 (3)	0.85653 (12)	0.23844 (9)	0.0294 (5)	
O2	0.0714 (2)	0.99090 (12)	0.32145 (9)	0.0256 (5)	
O3	−0.1713 (3)	1.05050 (13)	0.38002 (10)	0.0373 (6)	
O4	0.1377 (3)	1.03864 (14)	0.43763 (10)	0.0374 (6)	
C1	0.3544 (4)	0.99010 (16)	0.27833 (12)	0.0195 (6)	
C2	0.2189 (4)	1.03558 (17)	0.30114 (12)	0.0223 (6)	
C3	0.2174 (4)	1.12042 (17)	0.30137 (13)	0.0270 (7)	
H3	0.1209	1.1497	0.3181	0.032*	
C4	0.3583 (4)	1.15977 (17)	0.27698 (13)	0.0270 (7)	
H4	0.3617	1.2174	0.2781	0.032*	
C5	0.5000	1.1169 (2)	0.2500	0.0226 (9)	
C6	0.5000	1.0305 (2)	0.2500	0.0195 (8)	
C7	0.3417 (4)	0.89907 (17)	0.28618 (13)	0.0219 (6)	
C8	0.3937 (4)	0.86527 (16)	0.35419 (13)	0.0213 (6)	
C9	0.3690 (4)	0.78286 (17)	0.36579 (14)	0.0304 (7)	
H9	0.3199	0.7488	0.3301	0.036*	
C10	0.4155 (5)	0.75000 (19)	0.42896 (15)	0.0388 (8)	
H10	0.3986	0.6938	0.4372	0.047*	
C11	0.4865 (4)	0.8008 (2)	0.47904 (15)	0.0351 (8)	
C12	0.5127 (4)	0.88178 (19)	0.46988 (14)	0.0299 (7)	
H12	0.5618	0.9151	0.5060	0.036*	
C13	0.4663 (4)	0.91447 (17)	0.40671 (13)	0.0247 (6)	
H13	0.4841	0.9707	0.3993	0.030*	
C14	−0.0504 (4)	0.90554 (19)	0.41094 (15)	0.0326 (7)	
H14A	0.0650	0.8761	0.4204	0.039*	
H14B	−0.1230	0.8803	0.3727	0.039*	
H14C	−0.1182	0.9034	0.4503	0.039*	

### Atomic displacement parameters (Å^2^)

**Table:**

	*U*^11^	*U*^22^	*U*^33^	*U*^12^	*U*^13^	*U*^23^
S1	0.0244 (4)	0.0338 (4)	0.0201 (4)	−0.0002 (3)	0.0045 (3)	−0.0043 (3)
F1	0.0860 (17)	0.0496 (13)	0.0301 (11)	−0.0009 (11)	−0.0096 (10)	0.0191 (9)
O1	0.0402 (13)	0.0252 (11)	0.0221 (11)	−0.0055 (9)	0.0005 (9)	−0.0037 (9)
O2	0.0245 (11)	0.0340 (12)	0.0192 (10)	−0.0037 (9)	0.0057 (8)	−0.0036 (8)
O3	0.0278 (12)	0.0429 (14)	0.0423 (13)	0.0088 (10)	0.0089 (10)	−0.0026 (10)
O4	0.0337 (13)	0.0553 (15)	0.0230 (11)	−0.0083 (11)	0.0018 (9)	−0.0107 (10)
C1	0.0243 (14)	0.0216 (14)	0.0119 (13)	−0.0008 (12)	−0.0008 (10)	−0.0005 (10)
C2	0.0225 (15)	0.0302 (16)	0.0147 (14)	0.0001 (12)	0.0039 (11)	0.0012 (11)
C3	0.0312 (17)	0.0274 (16)	0.0225 (15)	0.0098 (13)	0.0027 (12)	−0.0010 (12)
C4	0.0344 (17)	0.0197 (15)	0.0256 (16)	0.0048 (13)	−0.0027 (13)	0.0013 (12)
C5	0.029 (2)	0.019 (2)	0.018 (2)	0.000	−0.0023 (16)	0.000
C6	0.023 (2)	0.019 (2)	0.0150 (19)	0.000	−0.0042 (15)	0.000
C7	0.0201 (15)	0.0236 (15)	0.0227 (15)	−0.0013 (12)	0.0054 (11)	−0.0005 (12)
C8	0.0215 (15)	0.0227 (15)	0.0204 (14)	−0.0007 (12)	0.0052 (11)	−0.0015 (11)
C9	0.0402 (19)	0.0229 (16)	0.0277 (17)	−0.0020 (14)	0.0019 (14)	−0.0018 (12)
C10	0.058 (2)	0.0225 (17)	0.0354 (19)	0.0004 (16)	0.0031 (16)	0.0061 (14)
C11	0.040 (2)	0.040 (2)	0.0248 (17)	0.0024 (15)	0.0031 (14)	0.0109 (14)
C12	0.0302 (17)	0.0371 (19)	0.0215 (15)	−0.0033 (14)	−0.0021 (12)	−0.0007 (13)
C13	0.0252 (16)	0.0214 (15)	0.0271 (16)	−0.0017 (12)	0.0014 (12)	0.0023 (12)
C14	0.0346 (18)	0.0398 (19)	0.0246 (16)	0.0004 (14)	0.0076 (13)	0.0011 (13)

### Geometric parameters (Å, º)

**Table:**

S1—O3	1.421 (2)	C5—C6	1.422 (5)
S1—O4	1.430 (2)	C6—C1^i^	1.432 (3)
S1—O2	1.6040 (19)	C7—C8	1.486 (4)
S1—C14	1.735 (3)	C8—C13	1.391 (4)
F1—C11	1.357 (3)	C8—C9	1.392 (4)
O1—C7	1.222 (3)	C9—C10	1.388 (4)
O2—C2	1.409 (3)	C9—H9	0.9500
C1—C2	1.366 (4)	C10—C11	1.368 (4)
C1—C6	1.432 (3)	C10—H10	0.9500
C1—C7	1.511 (4)	C11—C12	1.363 (4)
C2—C3	1.397 (4)	C12—C13	1.387 (4)
C3—C4	1.359 (4)	C12—H12	0.9500
C3—H3	0.9500	C13—H13	0.9500
C4—C5	1.416 (3)	C14—H14A	0.9800
C4—H4	0.9500	C14—H14B	0.9800
C5—C4^i^	1.416 (3)	C14—H14C	0.9800
			
O3—S1—O4	118.60 (14)	O1—C7—C1	120.4 (2)
O3—S1—O2	108.01 (12)	C8—C7—C1	117.0 (2)
O4—S1—O2	108.22 (12)	C13—C8—C9	119.2 (2)
O3—S1—C14	110.61 (15)	C13—C8—C7	121.3 (2)
O4—S1—C14	111.33 (14)	C9—C8—C7	119.5 (2)
O2—S1—C14	97.98 (12)	C10—C9—C8	120.6 (3)
C2—O2—S1	122.32 (16)	C10—C9—H9	119.7
C2—C1—C6	119.0 (3)	C8—C9—H9	119.7
C2—C1—C7	116.9 (2)	C11—C10—C9	118.1 (3)
C6—C1—C7	124.1 (2)	C11—C10—H10	120.9
C1—C2—C3	123.8 (3)	C9—C10—H10	120.9
C1—C2—O2	115.2 (2)	F1—C11—C12	118.3 (3)
C3—C2—O2	121.0 (2)	F1—C11—C10	118.5 (3)
C4—C3—C2	118.0 (3)	C12—C11—C10	123.2 (3)
C4—C3—H3	121.0	C11—C12—C13	118.6 (3)
C2—C3—H3	121.0	C11—C12—H12	120.7
C3—C4—C5	121.6 (3)	C13—C12—H12	120.7
C3—C4—H4	119.2	C12—C13—C8	120.3 (3)
C5—C4—H4	119.2	C12—C13—H13	119.8
C4—C5—C4^i^	120.2 (4)	C8—C13—H13	119.8
C4—C5—C6	119.90 (18)	S1—C14—H14A	109.5
C4^i^—C5—C6	119.90 (18)	S1—C14—H14B	109.5
C5—C6—C1	117.70 (17)	H14A—C14—H14B	109.5
C5—C6—C1^i^	117.70 (17)	S1—C14—H14C	109.5
C1—C6—C1^i^	124.6 (3)	H14A—C14—H14C	109.5
O1—C7—C8	122.5 (2)	H14B—C14—H14C	109.5
			
O3—S1—O2—C2	103.7 (2)	C2—C1—C6—C1^i^	−177.1 (3)
O4—S1—O2—C2	−25.9 (2)	C7—C1—C6—C1^i^	4.16 (18)
C14—S1—O2—C2	−141.5 (2)	C2—C1—C7—O1	101.4 (3)
C6—C1—C2—C3	−3.0 (4)	C6—C1—C7—O1	−79.9 (3)
C7—C1—C2—C3	175.8 (2)	C2—C1—C7—C8	−76.7 (3)
C6—C1—C2—O2	173.41 (18)	C6—C1—C7—C8	102.0 (3)
C7—C1—C2—O2	−7.8 (3)	O1—C7—C8—C13	176.3 (3)
S1—O2—C2—C1	130.4 (2)	C1—C7—C8—C13	−5.6 (4)
S1—O2—C2—C3	−53.0 (3)	O1—C7—C8—C9	−3.9 (4)
C1—C2—C3—C4	0.5 (4)	C1—C7—C8—C9	174.2 (3)
O2—C2—C3—C4	−175.7 (2)	C13—C8—C9—C10	0.2 (4)
C2—C3—C4—C5	2.0 (4)	C7—C8—C9—C10	−179.6 (3)
C3—C4—C5—C4^i^	178.0 (3)	C8—C9—C10—C11	−0.2 (5)
C3—C4—C5—C6	−2.0 (3)	C9—C10—C11—F1	−179.5 (3)
C4—C5—C6—C1	−0.47 (16)	C9—C10—C11—C12	0.3 (5)
C4^i^—C5—C6—C1	179.53 (16)	F1—C11—C12—C13	179.5 (3)
C4—C5—C6—C1^i^	179.53 (16)	C10—C11—C12—C13	−0.4 (5)
C4^i^—C5—C6—C1^i^	−0.47 (16)	C11—C12—C13—C8	0.3 (4)
C2—C1—C6—C5	2.9 (3)	C9—C8—C13—C12	−0.3 (4)
C7—C1—C6—C5	−175.84 (18)	C7—C8—C13—C12	179.6 (2)

Symmetry code: (i) −*x*+1, *y*, −*z*+1/2.

### Hydrogen-bond geometry (Å, º)

Cg is the centroid of the C8–C13 ring.

**Table:**

*D*—H···*A*	*D*—H	H···*A*	*D*···*A*	*D*—H···*A*
C14—H14*A*···*Cg*	0.98	2.87	3.805 (4)	160
C14—H14*B*···O1^ii^	0.98	2.45	3.399 (4)	163
C14—H14*C*···O4^iii^	0.98	2.46	3.304 (4)	144
C12—H12···O4^iv^	0.95	2.50	3.285 (4)	140
C4—H4···O1^v^	0.95	2.54	3.415 (4)	153

Symmetry codes: (ii) −*x*, *y*, −*z*+1/2; (iii) −*x*, −*y*+2, −*z*+1; (iv) −*x*+1, −*y*+2, −*z*+1; (v) −*x*+1/2, *y*+1/2, −*z*+1/2.
